# Analytical approach for determining beam profiles in water phantom of symmetric and asymmetric fields of wedged, blocked, and open photon beams

**DOI:** 10.1120/jacmp.v14i6.3918

**Published:** 2013-11-04

**Authors:** Mohamad Javad Tahmasebi Birgani, Nahid Chegeni, Shole Arvandi, Sasan Razmjoo Ghalaee, Mansoor Zabihzadeh, Davood Khezerloo

**Affiliations:** ^1^ Department of Radiation Therapy Golestan Hospital JondiShapour University of Medical Science Ahvaz Iran

**Keywords:** dose distribution, equivalent field, asymmetric field, irregular field, treatment planning

## Abstract

Nowadays, in most radiotherapy departments, the commercial treatment planning systems (TPS) used to calculate dose distributions needs to be verified; therefore, quick, easy‐to‐use, and low‐cost dose distribution algorithms are desirable to test and verify the performance of the TPS. In this paper, we put forth an analytical method to calculate the phantom scatter contribution and depth dose on the central axis based on the equivalent square concept. Then, this method was generalized to calculate the profiles at any depth and for several field shapes — regular or irregular fields—under symmetry and asymmetry photon beam conditions. Varian 2100 C/D and Siemens Primus Plus linacs with 6 and 18 MV photon beam were used for irradiations. Percentage depth doses (PDDs) were measured for a large number of square fields for both energies and for 45° wedge, which were employed to obtain the profiles in any depth. To assess the accuracy of the calculated profiles, several profile measurements were carried out for some treatment fields. The calculated and measured profiles were compared by gamma‐index calculation. All γ–index calculations were based on a 3% dose criterion and a 3 mm dose‐to‐agreement (DTA) acceptance criterion. The γ values were less than 1 at most points. However, the maximum γ observed was about 1.10 in the penumbra region in most fields and in the central area for the asymmetric fields. This analytical approach provides a generally quick and fairly accurate algorithm to calculate dose distribution for some treatment fields in conventional radiotherapy.

PACS number: 87.10.Ca

## I. INTRODUCTION

The fundamental physical quantity of interest for relating radiation treatment to its outcome is the absorbed dose. Furthermore, a significant component of the absorbed dose at a point is due to the scattering of the primary beam; therefore, it is essential to include the correct amount of scattering in any dose calculation algorithm used in treatment planning system (TPS). These algorithms are correction‐based or model‐ (or convolution–superposition‐) based. Correction‐based algorithms use parameters of dose measured in water phantom and correct the data to apply to the patient's specific situation. This requires the percentage depth dose (PDD) for a number of square fields, a set of profiles for a number of square fields measured at some standard depths, and phantom scatter factor curve at reference depth as a function of the field size of the square fields.^(^ ^1^ ^,^ ^2^ ^)^ Model‐based algorithms directly compute the dose to the patient by modeling the beam and interactions in the patient, which require some measurements to set parameters and verify the model. Monte Carlo methods, implemented to mimic the basic processes in a straightforward way, have served many purposes in medical physics. However, they have not yet become suitable for routine treatment planning of photon beams due to their huge requirement for CPU time.^(3)^ Consequently, analytical methods which would be reliable within an acceptable limit of error and for reasonable range of parameters to calculate PDD and profile at any depth are increasingly desirable. Increased requirements on standards for safety and quality assurance during treatment have also emphasized the important role of simple dose calculation methods for independent checks of the output from treatment planning systems.

For a given energy spectrum incident on a homogeneous medium and assuming lateral electronic equilibrium, the primary component of central axis depth dose for any field shape will be the same, and only differences in the scatter component will affect the final shape of the central axis depth dose; therefore, the concept of equivalent field size based on the separation of the primary and scatter radiation was proposed by Day and Aird.^(4)^ For regular fields, tabulated data was presented by Day and Aird, or some empirical formulas by fitting were carried out, such as the equal area‐to‐perimeter ratio (A/P).^(5)^

Various methods have been used for the prediction of off‐axis ratio for a symmetric open field. Fermi‐Dirac distribution function suggested by Kornelson and Young^(6)^ and Wood‐Saxon term applied by Pal et al.^(7)^ represent the off‐axis ratio (OAR) in the SAD and SSD techniques, respectively. Usually these methods need data fitting at several depths.

In the 1990s, some studies have addressed asymmetric and wedged asymmetric fields using symmetric field data.^8^, ^9^, ^10^, ^11^ The method based on work by Thomas and Thomas^(10)^ generates asymmetric field profiles by computing the off‐center ratio (OCR) of the asymmetric field while using output factors and PDDs of the equivalent symmetric field. A second method, proposed by Kwa et al.,^(12)^ applies to the situation where only one of the independent jaws is closed down to form an asymmetric field of smaller width or length than the original symmetric field. This method uses the original symmetric field profile corrected point‐by‐point by a correction factor.

Accordingly, we put forward a correction‐based dose calculation algorithm based on equivalent field concept for the fixed source‐to‐surface distance (SSD) formalism and develop proper analytical expression which could equip the computer with this formula for direct calculation of the profiles at any depth for some fields (e.g., symmetric or asymmetric field with or without wedge and blocks).

This paper introduces an analytical method to calculate the equivalent field in regard to the central axis, first. It will then follow by generalizing this method to a more general case in which the equivalent field is calculated concerning off‐axis points. Then, some profiles will be measured to set the imperial correction factors required to the calculations. Finally, the calculated and measured profiles will be compared for some practical fields.

### A. Theoretical background

#### A.1 Central axis percentage depth dose (PDD)

In this study, to calculate PDD for a custom field, the equivalent field method is used. A set of PDD is tabulated for a number of square fields measured along the central beam axis for both energies 6 and 18 MV and for open fields and every wedge angle separately. For any other square field size calculated, one can interpolate from the table of PDDs or utilize semiempirical equations for PDD depending on field size and depth.^(^ ^13^ ^,^ ^14^ ^)^

For any fixed point at fixed depth on the central axis in the medium, the primary component of the dose will be the same for all fields. It therefore follows that equivalency between standard and nonstandard fields is determined by the equal dose contribution along the central axis from scattered photons for the two fields.^(4)^ The method used to determine the scatter contribution the equivalent field in this study is further explained.

Consider a reference plane normal to the central axis, placed at the fixed source‐to‐surface distance (e.g., SSD=100cm). The field bound generated by the collimators can be projected over this plane as X1, X2, Y1, and Y2. The origin (e.g., x=0,y=0) is on the central axis. Suppose that there is a parallel beam striking the surface of the reference plane. Hence, each surface element (ds=dx·dy) on that plane acts as a source of scattered radiation. The amount of scatter radiation reaching the central axis is inversely proportional to the square of the distance between surface elements and the origin; therefore, an asymmetric irregular field (e.g., an asymmetric field with some shielded parts by cerrobend blocks or multileaf collimators (MLC)) to circular field equivalence at the central axis is calculated using the following equation:

(1) $$\int_{Y2}^{Y1}\int_{X2}^{X1}\frac{dxdy}{x^{2}+y^{2}}-\left((1-T_{b})\times \iint_{shielde\text{dp}arts}\frac{dxdy}{x^{2}+y^{2}}\right)=\iint_{equivalen\text{tc}ircle}\frac{dxdy}{x^{2}+y^{2}},$$

where $T_{b}=e^{-\mu_{b}t_{b}}$, which shows the transmission of the blocks or the leaves of MLC in which $\mu_{b}$ is the block attenuation coefficient and $t_{b}$ is its thickness. $\left(1-T_{b}\right)$ shows the fraction of primary photons absorbed by the block when they are passing through it. Therefore, in the absence of the block, $T_{b}$ will be 1 and the second term on the left side will be removed. It should be noted the attenuation of the water phantom using the factor of $\text{exp}(-\mu_{\text{water}}\cdot \sqrt{x^{2}+y^{2}})$ was considered on both sides of Eq. (1), where $\mu_{\text{water}}$ was the water phantom attenuation coefficient for scattered photons.^(15)^ However, because of its negligible effect, the attenuation of the water has been eliminated.

Let us use a polar system and eliminate the small area at the origin of coordinate with radius e (refers to the ion chamber radius) to overcome singularity; therefore, by dividing the field into four parts regarding the origin and integrating both sides of Eq. (1) with some mathematic operations, it can be rewritten as:

(2) $$\begin{array}{l} R_{eq}=\sqrt{Y1.Y2}{\left(\frac{X1}{Y1}\right)}^{{\text{tan}}^{-1}(\frac{Y1}{X1})/2\pi }{\left(\frac{X1}{Y2}\right)}^{{\text{tan}}^{-1}(\frac{Y2}{X1})/2\pi }{\left(\frac{X2}{Y1}\right)}^{{\text{tan}}^{-1}(\frac{Y1}{X2})/2\pi }{\left(\frac{X2}{Y2}\right)}^{{\text{tan}}^{-1}(\frac{Y2}{X2})/2\pi }\times \\ \text{exp}\left(\frac{-1}{2\pi }\left(\int_{{\text{tan}}^{-1}(\frac{Y1}{X1})-\frac{\pi }{2}}^{{\text{tan}}^{-1}(\frac{Y1}{X1})}\text{ln}(\text{cos}\theta )d\theta +\int_{{\text{tan}}^{-1}(\frac{Y2}{X1})-\frac{\pi }{2}}^{{\text{tan}}^{-1}(\frac{Y2}{X1})}\text{ln}(\text{cos}\theta )d\theta +\int_{{\text{tan}}^{-1}(\frac{Y1}{X2})-\frac{\pi }{2}}^{{\text{tan}}^{-1}(\frac{Y1}{X2})}\text{ln}(\text{cos}\theta )d\theta +\int_{{\text{tan}}^{-1}(\frac{Y2}{X2})-\frac{\pi }{2}}^{{\text{tan}}^{-1}(\frac{Y2}{X2})}\text{ln}(\text{cos}\theta )d\theta -(1-T)\sum_{i=1}^{n}\int_{shield}\int_{i^{th}}\frac{d\theta dr}{r}\right)\right) \end{array}$$

where the exponential parts are computable by numerical calculation using MATLAB software (The MathWorks, Natick, MA). As expected, e will be eliminated because it is present on both sides of Eq. (2). As a special case, the circular field equivalent to a square field is obtained by substituting S (side of square) in Eq. (2):

(3) $$R_{eq}=\frac{S}{2}\text{exp}\left(-\frac{2}{\pi }\int_{-\pi /4}^{\pi /4}\text{ln}(\text{cos}(\theta ))d\theta \right)=\frac{S}{2}(1.116)$$

where the integral is equal to −0.1728 using numerical integration. After calculating the size of the field side, the percentage depth dose at any depth can be interpolated from data tabulated for the square fields.

#### A.2 Computation of profile

Dose distributions along the beam central axis give only part of the information required for an accurate dose description inside the patient. Dose distributions in 2D and 3D are determined by central axis data in conjunction with off‐axis dose profiles. Megavoltage X‐ray beam profiles consist of three distinct regions: central, penumbra, and umbra. The central area represents the central portion of the profile extending from the beam's central axis to within 1–1.5 cm from the geometric field edges of the beam (e g., the 50% dose level points on the beam profile). In the penumbral region of the dose profile, the dose changes rapidly and depends also on the field defining collimators, the finite size of the focal spot (source size), and the lateral electronic disequilibrium. Umbra is the area outside the radiation field, far removed from the field edges. The dose in this district is generally low and results from radiation transmitted through the collimator and head shielding.^(16)^ The flattening filter also produces a differential hardening across the transverse direction of the beam which results in off‐axis peaking at a depth shallower than 10 cm. However, this model does not include the variations in off‐axis beam quality affected by a flattening filter.

**Figure 1:**
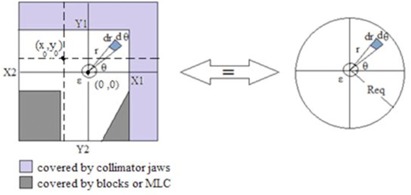
An asymmetric field with shielded parts by cerrobend blocks or MLC is equivalent to the circular field with radius $R_{\text{eq}}$, both projected on the phantom surface. *ε* is the ion chamber radius at SSD=100cm. The points (0, 0) or $\left(x_{0},\;y_{0}\right)$ illustrate the central axis of the beam and any point in the irradiated field, respectively.

**Figure 2:**
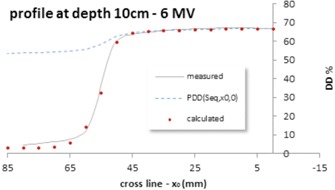
Profiles measured (solid) and calculated (circle) at depth 10 cm and 6 MV energy. $\text{PDD}(S_{\text{eq}},x_{0},0)$ (dash) shows PDDs on the central axis extracted from the data stored in the PDD table with regard to the equivalent square calculated from Eq. (5) for the point $\left(x_{0},\;0\right)$.

Consider a symmetric open field, regardless of the differential hardening effect of the flattening filter in the central region, the farther from the central axis of the radiation field, the less amount of phantom scatter; therefore, to calculate the amount of the phantom scatter reaching the point $\left(x_{0},y_{0}\right)$, the equivalent square regarding $\left(x_{0},y_{0}\right)$ is used (Fig. 1). Thus the total amount of the scattered radiation from the irradiated field reached to $\left(x_{0},y_{0}\right)$ is considered to be equal to the scattered radiation from an equivalent square field to the central axis and the side of this square is calculated. In this way, by dividing the field to four parts as regards the point $\left(x_{0},y_{0}\right)$, Eq. (2) can be modified as follows:

(4) $$\begin{array}{l} S_{eq(x_{0},y_{0})}=\frac{2\sqrt{\left(Y\;1-y_{0})\cdot (Y\;2-y_{0}\right)}}{1.116}{\left(\frac{X\;1-x_{0}}{Y\;1-y_{0}}\right)}^{{\text{tan}}^{-1}(\frac{Y\;1-y_{0}}{X\;1-x_{0}})/2\pi }{\left(\frac{X\;1-x_{0}}{Y\;2-y_{0}}\right)}^{{\text{tan}}^{-1}(\frac{Y\;2-y_{0}}{X\;1-x_{0}})/2\pi }{\left(\frac{X\;2-x_{0}}{Y\;1-y_{0}}\right)}^{{\text{tan}}^{-1}(\frac{Y\;1-y_{0}}{X\;2-x_{0}})/2\pi }{\left(\frac{X\;2-x_{0}}{Y\;2-y_{0}}\right)}^{{\text{tan}}^{-1}(\frac{Y\;2-y_{0}}{X\;2-x_{0}})/2\pi }\times \\ \text{exp}\left(\frac{-1}{2\pi }\left(\int_{{\text{tan}}^{-1}(\frac{Y1-y_{0}}{X1-x_{0}})-\frac{\pi }{2}}^{{\text{tan}}^{-1}(\frac{Y1-y_{0}}{X1-x_{0}})}\text{ln}(\text{cos}\theta )d\theta +\int_{{\text{tan}}^{-1}(\frac{Y2-y_{0}}{X1-x_{0}})-\frac{\pi }{2}}^{{\text{tan}}^{-1}(\frac{Y2-y_{0}}{X1-x_{0}})}\text{ln}(\text{cos}\theta )d\theta +\int_{{\text{tan}}^{-1}(\frac{Y1-y_{0}}{X2-x_{0}})-\frac{\pi }{2}}^{{\text{tan}}^{-1}(\frac{Y1-y_{0}}{X2-x_{0}})}\text{ln}(\text{cos}\theta )d\theta +\int_{{\text{tan}}^{-1}(\frac{Y2-y_{0}}{X2-x_{0}})-\frac{\pi }{2}}^{{\text{tan}}^{-1}(\frac{Y2-y_{0}}{X2-x_{0}})}\text{ln}(\text{cos}\theta )d\theta -\left((1-T)\times \iint_{shielde\text{dp}arts}\frac{drd\theta }{r}\right)\right)\right) \end{array}$$

where $S_{\text{eq}(x_{0},y_{0})}$ illustrates the calculated equivalent field regarding the point $\left(x_{0},y_{0}\right)$. It can also be shown that by substituting $x_{0}=0,\;y_{0}=0$ in Eq. (4), it will be transformed to Eq. (2). Now, this method is evaluated for the crossline profile $\left(x_{0},0\right)$ at depth 10 cm for 6 MV energy (Fig. 2). At first, $S_{\text{eq}(x_{0},0)}$ is calculated for some points on the crossline using Eq. (4). Subsequently, the percentage depth dose at depth 10 cm for 6 MV energy on the central axis can be interpolated for $S_{\text{eq}(x_{0},0)}$ from data tabulated for the square fields (see Fig. 2, $\text{PDD}(S_{\text{eq}},\;x_{0},\;0)$, dash line). As can be seen, it is in good agreement with the results of measurements up to 1.5 cm from the beam edge; therefore, for points near the edge of the beam, $\text{PDD}(S_{\text{eq}},x_{0},0)$ should be corrected.

As previously mentioned, in the penumbra region, the dose changes rapidly around the geometric beam edge and creates a sigmoidal shape. Furthermore, the photon source has a Gaussian distribution (normal distribution). Accordingly, the 3D photon influences the distribution of the primary source in air, which can be calculated by analytical integration of the Gaussian functions leading to an error function.^(17)^ Therefore, to calculate the profile (Fig. 2 circle) at depth, d, $\text{PDD}(S_{\text{eq}},\;x_{0},\;0)$ should be multiplied by the correction factor ofjaws as follows:

(5) $$\text{calculated}\;\text{profile}\;(x_{0},d)=\text{PDD}(S_{\text{eq}},x_{0},0,d)\times {{\text{CF}}_{J}(x}_{0})$$

where ${\text{CF}}_{J,X1}(x_{0})$ is the correction factor of the X1 jaw at $x_{0}$ which consists of three regions: infield, near outfield (penumbra), and far outfield (umbra). In the near outfield, both the transmission of the jaw and the correction of the electronic disequilibrium play a role. However, in the far outfield, the transmission of the jaw just presents (see Eq. (6)).

(6) $$CF_{J,X1}(x_{0})=\{\begin{array}{ll} \frac{1}{2}\left(1-erf\left(\frac{(\left|x_{0}\right|-X1)M}{\sqrt{2\sigma_{in}^{2}}}\right)\right) & x_{0}<X1\;(\text{in}-\text{field})\\ \frac{1}{2}\left(1-erf\left(\frac{(\left|x_{0}\right|-X1)M}{\sqrt{2\sigma_{out}^{2}}}\right)\right)+C_{e.diseq}+T_{J} & x_{0}\geq X1\;(\text{near}\;\text{out}-\text{field},\;\text{penumbra})\\ \frac{1}{2}\left(1-erf\left(\frac{(\left|x_{0}\right|-X1)M}{\sqrt{2\sigma_{out}^{2}}}\right)\right)+T_{J} & x_{0}\gg X1\;(\text{far}\;\text{out}-\text{field},\;\text{umbra}) \end{array}$$

where $T_{J}$ is the transmission through the collimator jaws, $C_{e\cdot \text{diseq}}$ is an empirical correction factor for electronic disequilibrium, $\sigma_{\text{in}}^{2}$ and $\sigma_{\text{out}}^{2}$. are the variance of inside and outside of the field, respectively, which are determined empirically for any energy. Due to beam divergence, in order to draw in‐depth (d) profile, magnification (M=(SSD+d)/SSD) should be considered. The ${\text{CF}}_{J}$ for X2, Y1, and Y2 are defined as Eq. (6) similarly. Obviously, the empirical constant mentioned above is independent from the depth, and field size due to $\text{PDD}(S_{\text{eq}},\;x_{0},\;0,\;d)$ includes the effects of both of them.

In the wedged field case, due to beam hardening, $\text{PDD}(S_{\text{eq}},\;x_{0},\;0)$ should be extracted from the table of measured PDD for several wedged square fields. Also, the primary beam is attenuated due to the wedge thickness variation. Hence, the wedge correction factor for any point off‐axis is described as the ratio of primary beam attenuation at that point to the central axis, as follows:

(7) $$CF_{w}=e^{-\mu_{w}(t_{w}-t_{0,w})}$$

where $\mu_{w}$ is the wedge attenuation coefficient, $t_{w}$ and $t_{0,w}$ are the wedge thickness corresponding to the points $\left(x_{0},y_{0}\right)$ and (0,0), respectively. Finally, by multiplying Eq. (5) in Eq. (7), the calculated wedge profile is obtained.

(8) $$\text{calculated}\;\text{wedged}\;\text{profile}\;(x_{0},d)=\text{PDD}(S_{\text{eq}},x_{0},0,d)\times {\text{CF}}_{J}(x_{0})\times {\text{CF}}_{w}$$

It should be noted that ${\text{CF}}_{w}$ equals to one for the open field.

For irregular fields, the blocks act like the collimators with variable thickness. So Eq. (6) can be rewritten for the block correction factor:

(9) $$CF_{b}(x_{1})=\{\begin{array}{ll} \frac{1}{2}\left(1-erf\left(\frac{(\left|x_{0}\right|-x_{i})M}{\sqrt{2\sigma_{in}^{2}}}\right)\right) & x_{0}\;\text{outside}\;\text{the}\;\text{bloczk}\\ \frac{1}{2}\left(1-erf\left(\frac{(\left|x_{0}\right|-x_{i})M}{\sqrt{2\sigma_{out}^{2}}}\right)\right)+C_{e\;.diseq}+T_{b} & x_{0}\;\text{under}\;\text{the}\;\text{block} \end{array}$$

where $x_{i}$ represents the coordinate of the point on the edge of block projected on the phantom surface and the crossline profile passes through it. $T_{b}=e^{-\mu_{b}t_{b}}$ shows the block transmission where $\mu_{b}$ is the block attenuation coefficient and $t_{b}$ is its thickness. Eq. (9) can be used for MLC, where $x_{i}$ represents the coordinate of the leaves edge of MLC projected on the phantom surface and $T_{b}$ shows the leaves transmission.

## II. MATERIALS AND METHODS

Varian 2100 C/D and Siemens Primus Plus with 6 and 18 MV photon beam were used for measurements. The treatment units were equipped with independent jaws assigned to as Y1 and Y2 for the upper and X1 and X2 for the lower jaws. A Scanditronix blue phantom (Wellhofer, Germany) (50cm×50cm×50cm) with two 0.13 CC ionization chambers (IBA, Germany) were employed for the measurement. Omni‐AcceptPro 6.5 software (Wellhofer, Germany) was connected to the interface and utilized for collecting and recording data from two chambers.

At first, PDDs were measured for a large number of square fields for both energies and for 45° wedge which were employed for profiles in any depth in this model. Then, to access the accuracy of the model for profile prediction, several profiles were measured for some special treatment fields such as symmetric, asymmetric, wedged asymmetric, and irregular fields (shown in 3, 4, 5, 6, 7).

Several dose distribution comparison methods have been developed based on various combinations of doses and spatial acceptance tolerances, including the simple dose difference (DD) test and the distance‐to‐agreement (DTA) test.^(^ ^18^ ^,^ ^19^ ^)^ The gamma index calculation and modified dose difference (MDdiff) evaluation are dose comparison methods which produce a quantitative measure based on both dose and spatial criteria.^20^, ^21^, ^22^ In this paper, the μ–index evaluation was utilized which was accorded a 3% dose criterion and a 3 mm dose‐to‐agreement (DTA) acceptance criterion.

Finally, a homemade computer program was developed in MATLAB 7.14 on Windows platform to process the data quickly, to plot the profiles, and to compare the calculations with measurements (see Appendix).

## III. RESULTS

At first, to set the empirical correction factors, profiles were calculated for a series of estimated correction factors for a standard field 10 cm by 10 cm in depth 10 cm and both energies 6 and 18 MV. Then, the calculated profiles were compared with the measured profiles using γ‐index evaluation. This procedure was repeated for different correction factors until the best ones were selected (e.g., γ ≤ 1). The final correction factors were obtained as follows:

(10) $$\{\begin{array}{l} \sigma_{in}=0.30,\;\sigma_{out}=0.6,\;T_{J}=0.01,\;C_{e\;.diseq}=0.05\;6MV\\ \sigma_{in}=0.55,\;\sigma_{out}=0.6,\;T_{J}=0.01,\;C_{e\;.diseq}=0.05\;18MV \end{array}$$

**Figure 3:**
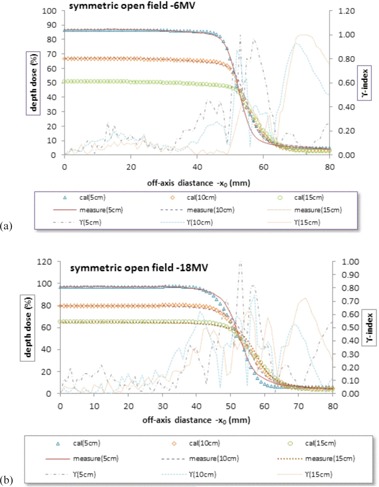
Measured and calculated crossline profiles and γ‐index (a) in depths 5, 10, and 15 cm for a symmetric open field 10 by 10 cm, Varian linac, and 6 MV energy; (b) measured and calculated crossline profiles and γ‐index in depths 5, 10, and 15 cm for a symmetric open field 10 by 10 cm, Varian linac, and 18 MV energy.

As shown in Figs. 3(a) and (b), these correction factors could be applied for depths 5 and 15 cm with acceptable γ‐index due to interpolating PDDs at 5 and 15 cm. In other words, the calculated correction factors for a given depth can be generalized to other depths. As can be seen in Figs. 3(a) and (b), the γ is less than 1 for most points. However, in the penumbra, γ‐index values represent larger numbers. The maximum γ values were 1.00, 0.93, and 1.00 for 6 MV. and 1.10, 0.75, and 0.73 for 18 MV at depth 5, 10, and 15 cm, respectively. It seems that the misalignment of the chamber axis plays role in the relatively high gamma values visible in the penumbral region. To clarify this, one can slightly shift the measured profile. Consequently, the γ values will be reduced in the penumbral region.

**Figure 4:**
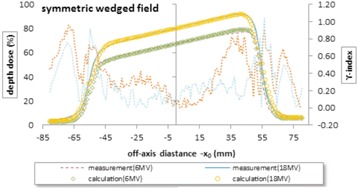
Measured and calculated crossline profiles and γ‐index in depth 10 cm for a symmetric wedged field 10 by 10 cm, wedge angle of 45°, Varian linac, and two energies 6 and 18 MV. The wedge attenuation coefficient $\mu_{W}$ was 0.5 and 0. 45 1/cm for energy of 6 and 18 MV, respectively.

In the second place, the calculated correction factors were used to the wedged field. Figure 4 shows the measured and calculated profiles in depth 10 cm for a symmetric wedged field 10 by 10 cm, wedge angle of 45°, Varian linac, and two energies 6 and 18 MV. As expected, the calculated profiles show good agreement with measurements by the γ less than 1 for most points, like the symmetric open field. Using the PDDs table regarding the wedged square fields will obviate the beam hardening which can affect the shape of profile at different depths. The maximum γ value was 0.96 and 1.05 for 6 MV and 18 MV, respectively.

**Figure 5:**
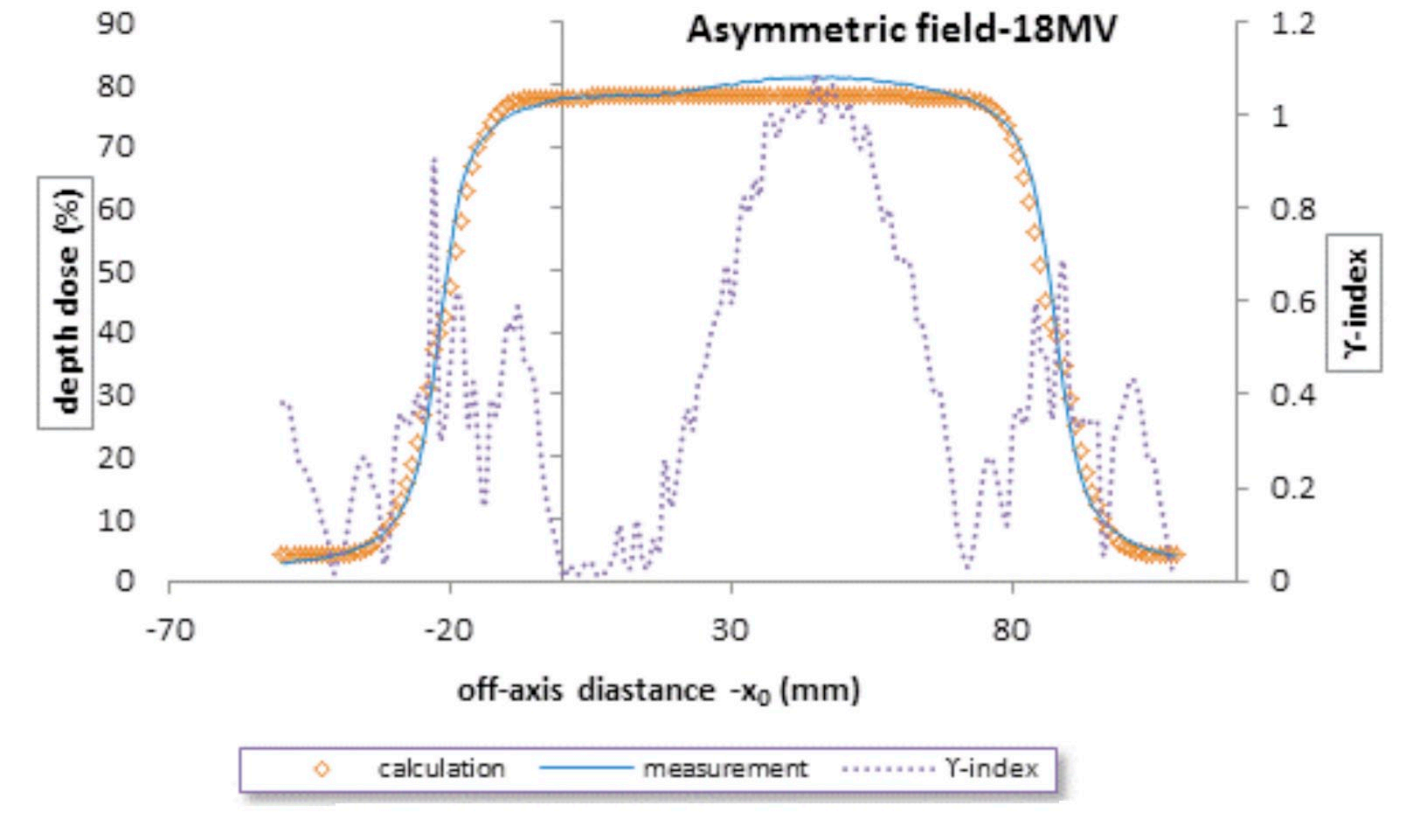
Measured and calculated the asymmetric profiles and γ‐index in depth 10 cm for asymmetric open field 10 by 10 cm with 3 cm offset at 100 cm SSD, Siemens linac, and 18 MV energy.

**Figure 6:**
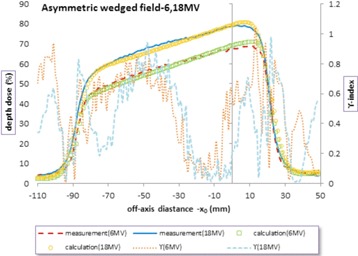
Measured and calculated asymmetric wedged profiles and γ‐index in depth 10 cm for field 10 by 10 cm with 3 cm offset at 100 cm SSD, wedge angle of 45°, Siemens linac, and two energies 6 and 18 MV. The wedge attenuation coefficient $\mu_{W}$ was 0.3 and 0.24 1/cm for energy of 6 and 18 MV, respectively.

For asymmetric fields, Fig. 5 represents the profiles measurement and calculation in depth 10 cm for an asymmetric open field 10 by 10 cm, with 3 cm offset at 100 cm SSD, Siemens linac, and 18 MV energy. As can be seen in Fig. 5, the asymmetric field has larger γ values in the central region, unlike the symmetric field. The maximum γ value was 1.09 in the central area. As shown in Fig. 6, the measured and calculated profiles were plotted in depth 10 cm for an asymmetric wedged field 10 by 10 cm with 3 cm offset, wedge angle of 45°, Siemens linac, and two energies 6 and 18 MV. As was mentioned for asymmetric field, here there are some points at which γ‐indexes are greater than 1 in the center district. This issue seems to be due to the lack of the flattening filter effect on the primary beam in this model, and this effect was larger in the profiles of Siemens linac.

**Figure 7:**
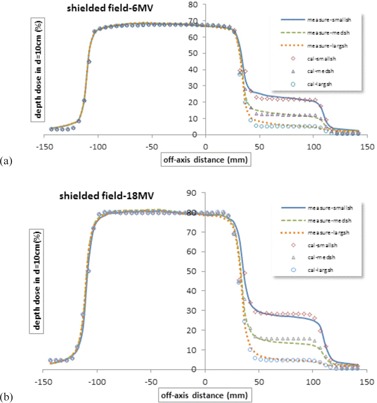
Measured and calculated symmetric shielded profiles and γ‐index (a) for three blocks dimensions of 7 by 7cm with 2.5, 4, and 7.9 cm thickness in the right corner of 10 by 20 cm field in depth 10 cm, Varian linac, and 6 MV energy, with the attenuation coefficient of block $\mu_{\text{block}}$ as 0.44 (1/cm); (b) measured and calculated symmetric shielded profiles and γ‐index for three blocks dimensions of 7 by 7 cm with 2.5, 4, and 7.9 cm thickness in the right corner of 10 by 20 cm field in depth 10 cm, Varian linac, and 18 MV energy, with the attenuation coefficient of block $\mu_{\text{block}}$ as 0.42 (1/cm).

The profiles for a rectangular field (10×20cm) with three blocks (7×7cm) with 2.5, 4, and 7.9 cm thickness in the right corner were evaluated in depth 10 cm for two energies 6 and 18 MV (Figs. 7(a) and 7(b)). Note that, to avoid clutter, these figures do not show the γ calculations. For more details, the mean γ‐index values and 1 standard deviation are given in Table 1 for all fields.

**Table 1:** Mean γ‐index and standard deviations of calculated and measured dose profiles for the fields used in this study

(Fig. 3(a), 3(b)) 10×10−open								
	*γ‐index–6MV*				*γ‐index – 18 MV*			
*Depth*	*Infield*		*Outfield*		*Infield*		*Outfield*	
*(cm)*	*AVR*	*STD*	*AVR*	*STD*	*AVR*	*STD*	*AVR*	*STD*
5	0.20	0.2	0.30	0.29	0.21	0.21	0.32	0.23
10	0.16	0.17	0.56	0.25	0.20	0.19	0.32	0.19
15	0.08	0.09	0.61	0.33	0.17	0.16	0.45	0.18
(Fig. 4) 10×10−wedge45°								
10	0.36	0.23	0.51	0.26	0.25	0.20	0.38	0.22
(Fig. 5) 10×10−open(offset3cm)								
10					0.25	0.22	0.47	0.31
(Fig. 6) 10×10−wedge45°(offset3cm)								
10	0.51	0.24	0.44	0.27	0.45	0.27	0.37	0.31
(Fig. 7(a)),7(b)) 10×20shielded								
10(small)	0.43	0.73	0.51	0.26	0.44	0.44	0.46	0.53
10(med)	0.41	0.64	0.50	0.29	0.64	0.77	0.36	0.16
10(large)	0.49	0.62	0.56	0.24	0.47	0.67	0.36	0.17

## IV. DISCUSSION & CONCLUSION

Today, the use of radiotherapy treatment planning systems (TPSs) is inevitable. Beam profile and PDD are the parameters used to verify the dose calculation algorithms of TPS; therefore, a patient‐independent model calculating beam profile and PDD can be used to minimize the number of measurements for verification processes.^(3)^

The algorithm used in this study calculates the beam profile in the water phantom by separation of the primary and the scatter beams, for irregularly blocked fields of open or wedged photon beam. The requirements are only the PDDs for several squares for each energy and wedge angle and some profiles to set the empirical correction factors. As can be seen in Table 1 and 3, 4, 5, 6, 7, the comparison with measurements using γ‐index shows that the accuracy of the calculated dose distributions fits well in a 3% error in low‐dose gradient region, except for asymmetric fields. In general, the primary dose rate at shallow depths in the phantom may actually increase at distances away from the central axis (called horns) as a result of flattening filter effect on the radiation beam.^10^, ^11^, ^12^ A flattening filter correction that depends on depth in a phantom and radial distance from the central axis is required to model the increase in dose rate away from the central beam axis that is not included in this paper. The comparison also shows an approximate agreement in a 3 mm isodose shift in the penumbra region. The dose near the edge of the beam changes rapidly and depends also on the field defining collimators, the source size, and the lateral electronic disequilibrium. Since the photon source has a Gaussian distribution (normal distribution), the dose falloff around the geometric beam edge is sigmoid in shape, for which an error function has been employed. However, considering the fact that the Gaussian distribution alone, like the models proposed by Pal et al.^(7)^ The Kornelsen and Batho^(23)^ model suffer from lack of electronic disequilibrium; therefore, a correction factor, ${\text{CF}}_{e,\text{diseq}}$, has been considered here.

The correction‐based algorithm^(^ ^1^ ^,^ ^2^ ^)^ and the previous methods mentioned^(^ ^7^ ^,^ ^23^ ^)^ need beam profile data for a large number of depths to predict the off‐axis ratio. In addition, a general problem with empirical scatter scaling techniques is that they are developed for open, not modulated beams, and there is a great need to improve these models to include effects from modulations.^(3)^ Nevertheless, the findings of the current study show the empirical correction factors are independent on depth. The depth independence is due to $\text{PDD}(S_{\text{eq}},\;x_{0},\;0,\;d)$ which includes the effects of the depth. In addition, the correction factor of jaw, ${\text{CF}}_{\text{JX}1}$, acts similarly not only for symmetric fields but also for asymmetric fields. The depth dose differences between symmetric and asymmetric field at any point are indicated by $\text{PDD}(S_{\text{eq}},\;x_{0},\;0,\;d)$ unlike the previous studies.^8^, ^9^, ^10^, ^11^, ^12^

This method is valuable due to the calculated profile in the presence of the blocks with variable thickness. The variable thickness shields can protect organ at risk inside the field. This means that organ at risk and normal tissue receive desirable tolerance dose, whereas the lethal dose is delivered to the tumor; further research should be done to investigate this in compensator‐based IMRT.

Consequently, a general algorithm was proposed to calculate the profile at any depth for symmetric and asymmetric, wedged or open photon fields. The advantages of this algorithm are depth independence, minimum measurement data requirements, quick‐run, low cost, easy‐to‐use, and fairly accurate. Moreover, this algorithm can be carried out to plot 2D or 3D isodose for multiple field treatment planning. A number of important caveats need to be considered about the calculation algorithm. First, it is limited to rectangular and triangular block shape. Second, it does not include the curvature of body surface and inhomogeneity. Finally, the flattening filter effect should be considered as another correction factor. It is recommended that further research be undertaken to solve these problems.

## ACKNOWLEDGMENTS

The authors thank the reviewers for their comments that help improve the manuscript. The authors would also like to thank Dr. Rahmani for his unsparing support. This study was funded by the office of Vice‐Chancellor for Research of Joundishapour University of Medical Science Ahvaz, and the Radiotherapy and Oncology Center of Ahvaz Golestan Hospital.

## APPENDICES

### Appendix A: The Procedure to Transition Eq. (2) from Eq. (1)

Using a polar system and eliminate the small area at the origin of coordinate with radius e and dividing the field into eight parts regarding the origin and integrating both sides of Eq. (1), it can be written as:

(A1) $$\left(\begin{array}{l} \int_{0}^{{\text{tan}}^{-1}(\frac{Y1}{X1})}\int_{\varepsilon }^{\frac{X1}{\text{cos}(\theta )}}\frac{drd\theta }{r}+\int_{{\text{tan}}^{-1}(\frac{Y1}{X1})}^{\frac{\pi }{2}}\int_{\varepsilon }^{\frac{Y1}{\text{sin}(\theta )}}\frac{drd\theta }{r}+\int_{0}^{{\text{tan}}^{-1}(\frac{Y1}{X2})}\int_{\varepsilon }^{\frac{X2}{\text{cos}(\theta )}}\frac{drd\theta }{r}+\int_{{\text{tan}}^{-1}(\frac{Y1}{X2})}^{\frac{\pi }{2}}\int_{\varepsilon }^{\frac{Y1}{\text{sin}(\theta )}}\frac{drd\theta }{r}+\int_{0}^{{\text{tan}}^{-1}(\frac{Y2}{X2})}\int_{\varepsilon }^{\frac{X2}{\text{cos}(\theta )}}\frac{drd\theta }{r}\\ +\int_{{\text{tan}}^{-1}(\frac{Y2}{X2})}^{\frac{\pi }{2}}\int_{\varepsilon }^{\frac{Y2}{\text{sin}(\theta )}}\frac{drd\theta }{r}+\int_{0}^{{\text{tan}}^{-1}(\frac{Y2}{X1})}\int_{\varepsilon }^{\frac{X1}{\text{cos}(\theta )}}\frac{drd\theta }{r}+\int_{{\text{tan}}^{-1}(\frac{Y2}{X1})}^{\frac{\pi }{2}}\int_{\varepsilon }^{\frac{Y2}{\text{sin}(\theta )}}\frac{drd\theta }{r}+\left((1-T_{b})\times \iint_{shiele\text{dp}arts}\frac{drd\theta }{r}\right) \end{array}\right)=\int_{0}^{2\pi }\int_{\varepsilon }^{R_{eq}}\frac{drd\theta }{r}$$

Integration with respect to r leads to natural logarithm as follows:

(A2) $$\left(\begin{array}{l} \int_{0}^{{\text{tan}}^{-1}(\frac{Y1}{X1})}\text{ln}(\frac{X1}{\varepsilon \text{cos}\theta })d\theta +\int_{{\text{tan}}^{-1}(\frac{Y1}{X1})}^{\frac{\pi }{2}}\text{ln}(\frac{Y1}{\varepsilon \text{sin}\theta })d\theta +\int_{0}^{{\text{tan}}^{-1}(\frac{Y1}{X2})}\text{ln}(\frac{X2}{\varepsilon \text{cos}\theta })d\theta +\int_{{\text{tan}}^{-1}(\frac{Y1}{X2})}^{\frac{\pi }{2}}\text{ln}(\frac{Y1}{\varepsilon \text{sin}\theta })d\theta +\int_{0}^{{\text{tan}}^{-1}(\frac{Y2}{X2})}\text{ln}(\frac{X1}{\varepsilon \text{cos}\theta })d\theta \\ +\int_{{\text{tan}}^{-1}(\frac{Y2}{X2})}^{\frac{\pi }{2}}\text{ln}(\frac{Y2}{\varepsilon \text{sin}\theta })d\theta +\int_{0}^{{\text{tan}}^{-1}(\frac{Y2}{X1})}\text{ln}(\frac{X1}{\varepsilon \text{cos}\theta })d\theta +\int_{{\text{tan}}^{-1}(\frac{Y2}{X2})}^{\frac{\pi }{2}}\text{ln}(\frac{Y2}{\varepsilon \text{sin}\theta })d\theta +\left((1-T_{b})\times \iint_{shiele\text{dp}arts}\frac{drd\theta }{r}\right) \end{array}\right)=\int_{0}^{2\pi }\int_{\varepsilon }^{R_{eq}}\frac{drd\theta }{r}$$

In the next step, integration respect to θ and by more simplification, it will be rearranged as Eq. (2).

The algorithm to calculate profile was as follows:

1) PDDs were measured for a number of square fields along the central beam axis, for open fields and 45° wedge and for both 6 and 18 MV energies separately. The PDDs were tabulated for desired interval (e.g., 0.5 cm) and stored in the MATLAB program.
2) A set of required parameters such as field size, wedge angle, and blocks properties (e.g., thickness, location and size), was fed to the calculation algorithm.
3) For open symmetric field, the equivalent square, $S_{\text{eq}(x_{0},0)}$ was calculated for any point placed on crossline $\left(x_{0},\;0\right)$ using Eq. (4).
4) To plot the profile at depth 10 cm, $\text{PDD}(S_{\text{eq}(x_{0},0)})$ was interpolated from stored data for both energies at depth 10 cm.
5) According 5, 6, profiles were calculated for a series of estimated correction factors for a standard field 10 cm by 10 cm in depth 10 cm and both energies 6 and 18 MV.
6) The γ‐index was calculated for every point; $\sigma_{\text{in}},\;\sigma_{\text{out}},\;T_{J}$ and ${\text{CF}}_{e,\text{diseq}}$ were changed until γ<1.
7) Finally, ${\text{CF}}_{J},{\text{CF}}_{w}$ and ${\text{CF}}_{b}$ were calculated to plot profiles for asymmetric, wedged, and irregular fields at any depth.

## Supporting information

Supplementary Material

Supplementary Material

Supplementary Material

Supplementary Material
