# Supplementary figures and images for: Analytical approach for determining beam profiles in water phantom of symmetric and asymmetric fields of wedged, blocked, and open photon beams
Source: J Appl Clin Med Phys. 2013 Nov 4;14(6):1–13. doi: 10.1120/jacmp.v14i6.3918 (PMC5714631; doi:10.1120/jacmp.v14i6.3918)

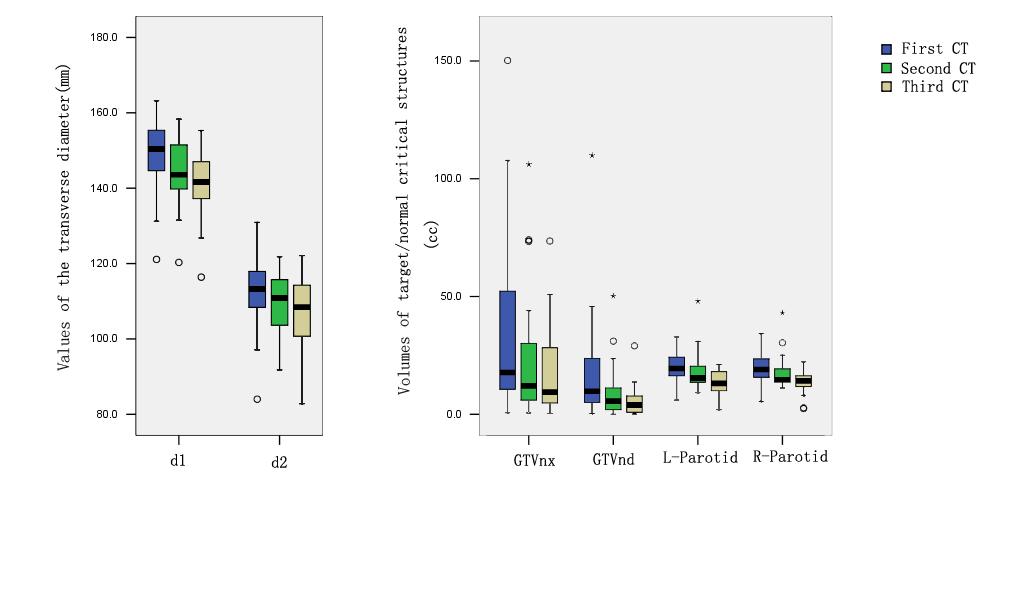

Supplement: Supplementary file 1 — Supplementary Material [file ACM2-14-001b-s001.JPG]

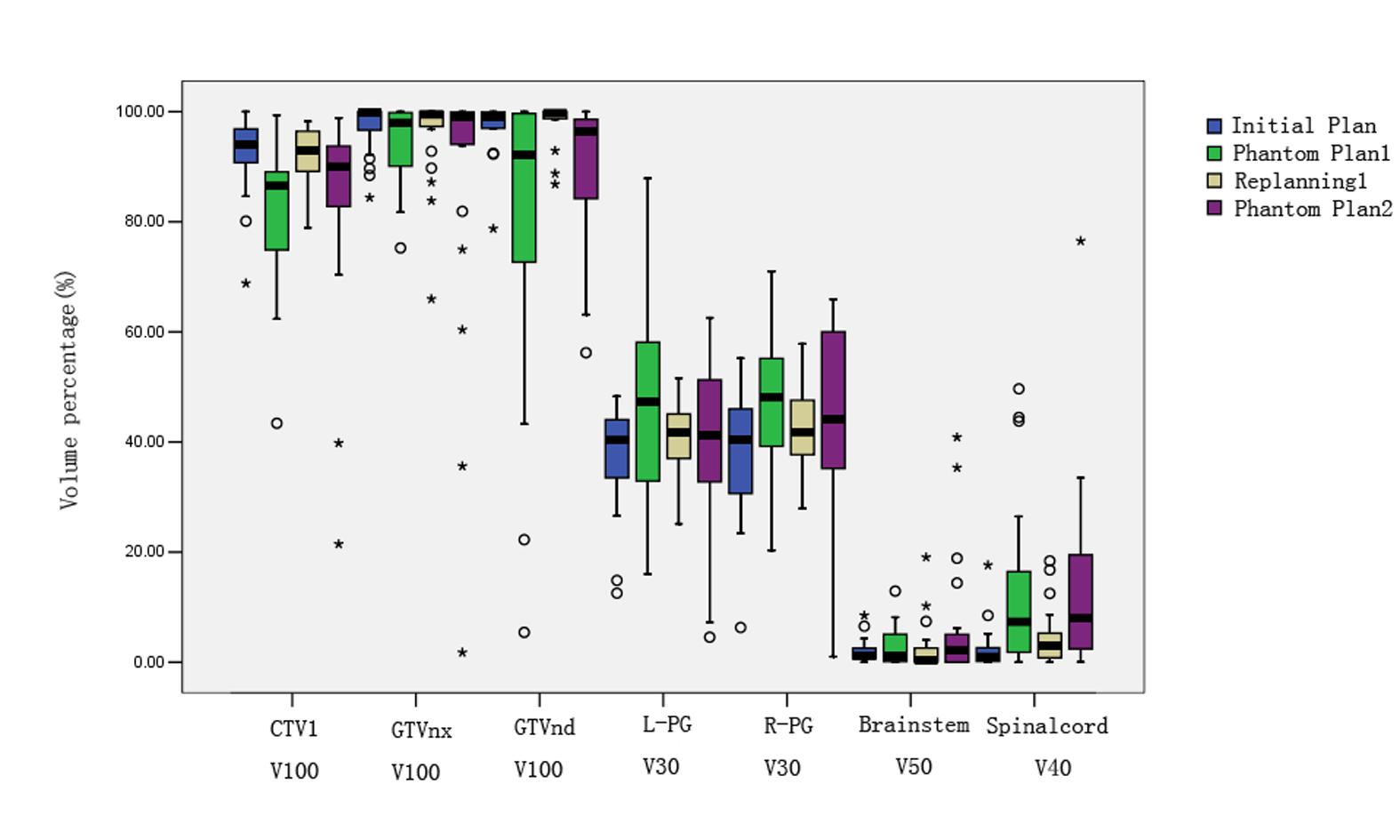

Supplement: Supplementary file 2 — Supplementary Material [file ACM2-14-001b-s002.JPG]

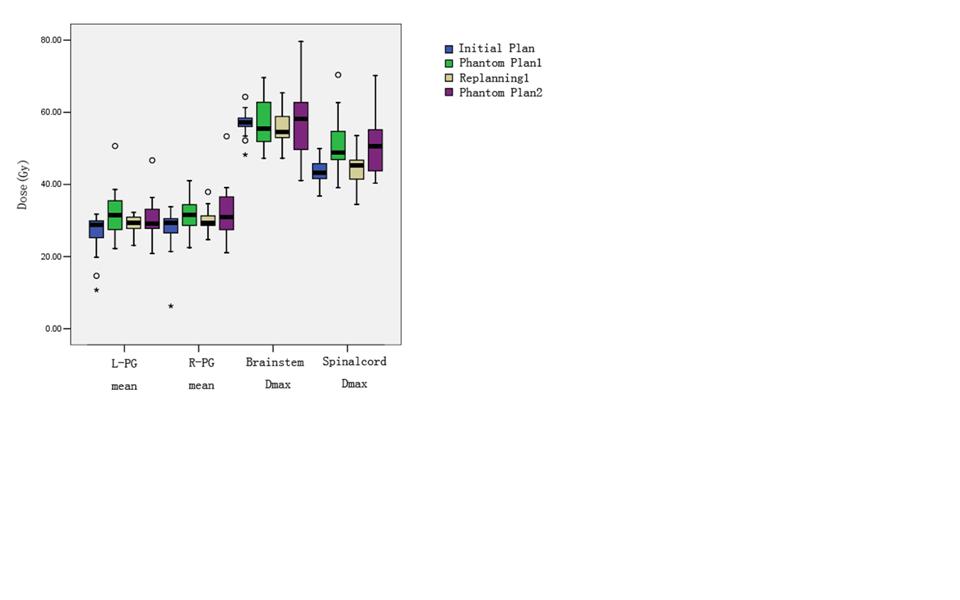

Supplement: Supplementary file 3 — Supplementary Material [file ACM2-14-001b-s003.JPG]
