# Supplementary material for: Analytical approach for determining beam profiles in water phantom of symmetric and asymmetric fields of wedged, blocked, and open photon beams
Source: J Appl Clin Med Phys. 2013 Nov 4;14(6):1–13. doi: 10.1120/jacmp.v14i6.3918 (PMC5714631; doi:10.1120/jacmp.v14i6.3918)
Supplement: Supplementary file 4 — Supplementary Material [file ACM2-14-001b-s004.doc]

**A comparison of anatomical and dosimetric variations in the first 15 fractions and between fractions 16 and 25 of intensity-modulated radiotherapy for nasopharyngeal carcinoma**

Haihua Yang, M.D.*^1, 2^, Yu Tu, Ph.D.^3^, Wei Wang, B.S.^1, 2^ , Wei Hu, M.D.^1, 2^, Weijun Ding, M.D.^1, 2^,

Changhui Yu, B.S.^1, 2^, Chao Zhou, M.D.^1, 2^

^1^Department of Radiation Oncology, ^2^Laboratory of Cellular and Molecular Radiation Oncology, Taizhou Hospital, Wenzhou Medical College, Taizhou, 317000, Zhejiang, China

^3^Jiangsu Provincial Key Laboratory of Radiation Medicine and Protection, School of Radiation Medicine and Protection, Medical College of Soochow University, Suzhou, 215123, Jiansu, China.

^*^Reprint requests to: Haihua Yang, M.D.

Department of Radiation Oncology, Taizhou Hospital, Wenzhou Medical College, Taizhou, 317000, Zhejiang, China.

Tel: +86-138-19639006; Fax: +86-576-85199876;

E-mail: [yhh93181@hotmail.com](mailto:yhh93181@hotmail.com)

Running title: Anatomical and dosimetric variations in different periods of IMRT for NPC

The abstract of the study has been presented at the 53^rd^ 2011 Joint AAPM/COMP Meeting (July 31 – August 4, Vancouver).

Conflict of interest: none.
